# Successful Percutaneous Retrieval of Embolized Left Atrial Appendage Occlusion Device From Left Ventricle

**DOI:** 10.1016/j.jaccas.2025.105902

**Published:** 2025-12-10

**Authors:** Nihar Jena, Boney Lapsiwala, Gayatri Bondi, Quang Dat Ha, Yash Verma, Prabhat Singh, Hari Naga Garapati, Sreekant Avula, Rameez Sayyed, Kirit Patel

**Affiliations:** a Department of Interventional Cardiology, Marshall University, Huntington, West Virginia, USA; b Department of Internal Medicine, Medical City Arlington, Arlington, Texas, USA; c Department of Cardiology, Trinity Health Oakland/Wayne State University, Pontiac, Michigan, USA; d Department of Nephrology, Kidney Specialists of South Texas, Corpus Christi, Texas, USA; e Department of Nephrology, Baptist Medical Center South, Montgomery, Alabama, USA; f Department of Diabetes and Metabolism, University of Minnesota, Minneapolis, Minnesota, USA

**Keywords:** atrial fibrillation, left atrial appendage occlusion device (LAAO), percutaneous, snare

## Abstract

**Background:**

The left atrial appendage occlusion (LAAO) device is a noninferior alternative to anticoagulation therapy in patients with atrial fibrillation. Device embolization is a rare but possible complication, often requiring surgical intervention specifically for left ventricular retrieval.

**Case Summary:**

An 80-year-old woman underwent LAAO device placement according to standard protocol, but the device embolized within a few hours. The embolized LAAO device was successfully retrieved percutaneously with a triple-loop snare using a combined transseptal and transarterial approach.

**Discussion:**

The percutaneous approach highlighted in the case offers a unique, minimally invasive method for retrieving the embolized LAAO device, thereby avoiding the need for open heart surgery.

**Conclusions:**

LAAO device embolization is a rare potential complication that requires monitoring after the procedure. Percutaneous retrieval using a triple-loop snare is a viable option.

## Graphical Abstract

**Figure:**
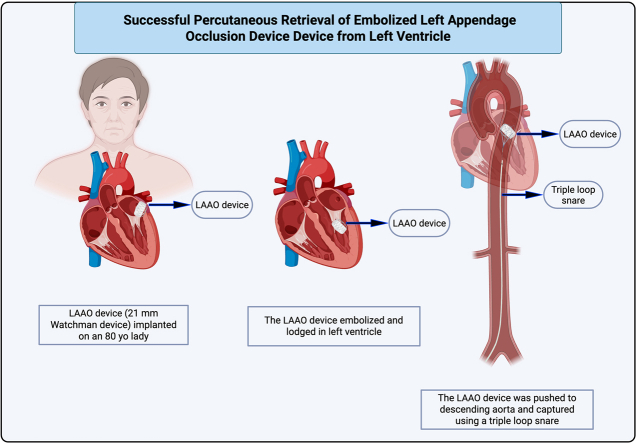

## History of Presentation

An 80-year-old woman was found to have frequent premature ventricular contractions on postoperative monitoring after uncomplicated 21-mm transseptal Watchman device (Boston Scientific) placement which met the PASS (position, anchoring, sizing, and sealing) criteria.^1^

**Take-Home Messages**

• High clinical suspicion and echocardiography should be used as a diagnostic modality for embolized LAAO devices.
• The surgical approach should be the first-line management plan for hemodynamically compromised patients with device embolization.
• Selective low-risk patients can undergo a percutaneous approach to remove the embolized device.

## Past Medical History

**Figure 1:**
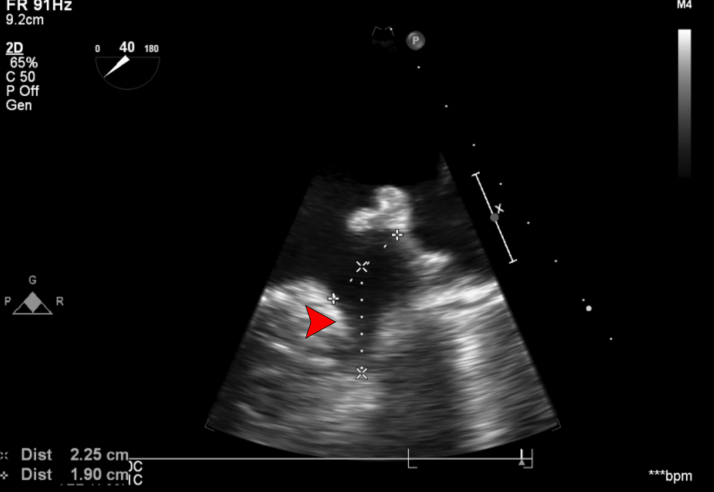
TEE Midesophageal Short-Axis View at 40° Showing the Ostial Diameter of 1.90 cm (+) and Depth of 2.25 cm (×) The red arrowhead indicates the left atrial appendage. TEE = transesophageal echocardiography.

The patient had chronic and persistent nonvalvular atrial fibrillation, a CHA_2_DS_2_-VASc score of 6 and a HAS-BLED score of 4, and was intolerant to oral anticoagulants owing to severe lower gastrointestinal bleeding. She had a prior multivessel percutaneous coronary intervention with preserved left ventricular (LV) function, diastolic heart failure (NYHA functional class III), moderate mitral regurgitation, and hypertension. Multidetector computed tomography angiogram and transesophageal echocardiography (TEE) had been performed to determine the size of the left atrial appendage occlusion (LAAO) device before the procedure. The appendage morphology was of the windsock type, with a single lobe having a maximum ostial diameter of 1.9 cm and a depth of 2.25 cm (Figure 1). The device was placed successfully, and a tug test was performed at the end.

## Investigations

**Figure 2:**
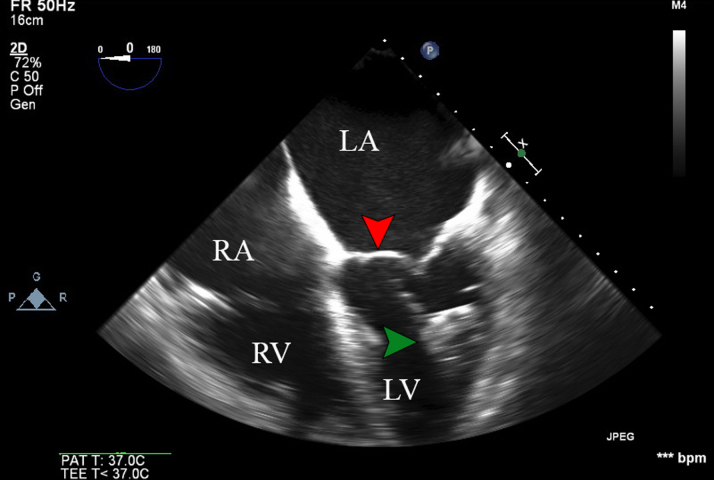
TEE Midesophageal 4-Chamber View Showing an Embolized Watchman Device (Green Arrowhead) Lodged in the Left Ventricle The red arrowhead indicates the mitral valve. LA = left atrium; LV = left ventricle; RA = right atrium; RV = right ventricle; TEE = transesophageal echocardiography.

The patient was monitored in the cardiac critical care unit, and she experienced frequent premature ventricular contractions. Transthoracic echocardiography revealed that the device had been embolized into the LV and wedged in the mitral subvalvular apparatus (Figure 2).

## Management

The patient was immediately taken to the cardiac catheterization laboratory in stable condition for attempted retrieval of the Watchman device using both anterograde and retrograde approaches. TEE confirmed that the LAAO device had migrated distally toward the papillary muscle and was wedged between the lateral wall and the papillary muscle (Figure 2).

Under ultrasound guidance, 8-F short sheaths were introduced into the right femoral artery and vein. The 8-F sheath in the right femoral vein was upgraded to a 55-cm Baylis sheath (Baylis Medical) to access the left atrium (LA) via transseptal puncture. A wire was advanced into the LA under TEE guidance. The LA opening pressure was recorded, with a mean pressure of approximately 10 to 15 mm Hg. Intravenous heparin was administered to maintain an activated clotting time of over 300 to 350 seconds. Simultaneously, a 16-F sheath was placed in the right femoral artery, with preclosure performed using Perclose sutures (Abbott Laboratories).

The Baylis sheath was then upgraded to a medium-curve DiRex sheath (Boston Scientific), which allowed for improved manipulation and access to the LV. A ThermoCool SmartTouch ablation catheter (Biosense Webster) was advanced through the DiRex sheath into the LV and was used to manipulate the device. The device was successfully freed from the lateral wall under fluoroscopic and TEE guidance and was allowed to migrate through the aortic valve into the descending aorta.

**Figure 3:**
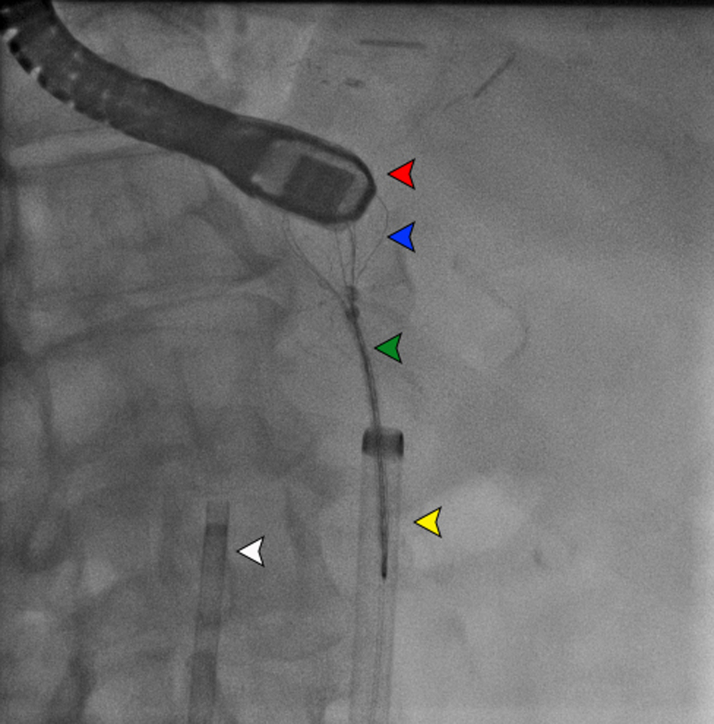
Fluoroscopic Image Showing Aortic Snare Removal of the Embolized Watchman Device The red arrowhead indicates the TEE probe; the blue arrowhead indicates the embolized Watchman device captured by snare; the green arrowhead indicates the triple-loop snare; the yellow arrowhead indicates the 16-F aortic sheath; and the white arrowhead indicates the ThermoCool ablation sheath. TEE = transesophageal echocardiography.

**Figure 4:**
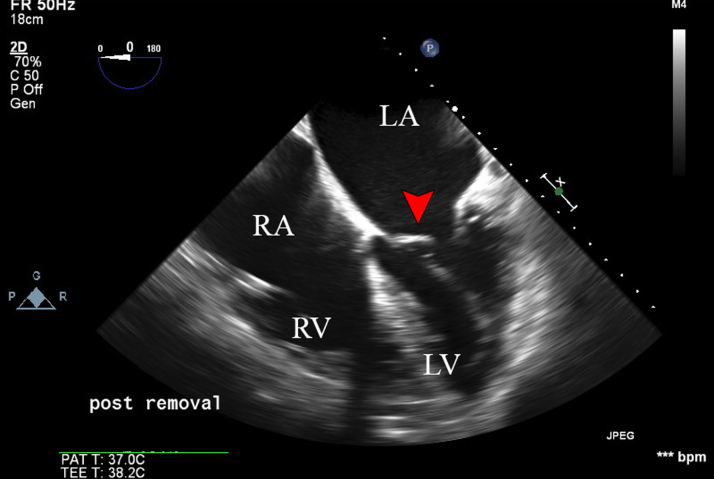
TEE Midesophageal 4-Chamber View After Removal of the Embolized Device The red arrowhead indicates the mitral valve. Abbreviations as in Figure 2.

The device was subsequently captured using the EN Snare (triple-loop snare, Merit Medical) and was safely explanted through the 16-F long sheath (Figure 3). TEE showed no pericardial effusion and no valvular regurgitation (Figure 4). The patient recovered from anesthesia without neurological deficits. She had an uneventful overnight recovery and was discharged the following day on aspirin 81 mg and apixaban 5 mg twice daily. The timeline of the case is shown in Table 1. The devices used during the procedure are detailed in Table 2.

**Table 1:** Timeline of the Case

Time	Events
Day of procedure/day 1	An 80-year-old woman with atrial fibrillation presented for elective LAAO device placement. The device size was predetermined by TEE and CTA.
Day 1	Within a few hours, the device embolized to the left ventricle, which was visualized by TTE.
Procedure	A transseptal and transarterial approach with a triple-loop snare was used to capture the device from the descending aorta.
Postprocedure	The patient was evaluated for 2 days and discharged home.
Follow-up, 1 wk, 4 wk, and 6 mo	She was maintained on anticoagulation with careful monitoring of her hemoglobin levels.
8 mo	The patient underwent Amplatzer Amulet device placement with a successful outcome.
Follow-up, 1 wk and 45 d	Clinically well with no complications. TEE revealed a sealed device with no evidence of thrombus.

CTA = computed tomography angiogram; LAAO = left atrial appendage occlusion; TEE = transesophageal echocardiography.

**Table 2:** List of Devices Used in the Case

Device Used	Manufacturer	Role
21-mm Watchman device	Boston Scientific	Initial LAAO implant
55-cm Baylis sheath	Baylis Medical	Transseptal access to the left atrium
Medium-curve DiRex sheath	Boston Scientific	Access to the left ventricle
ThermoCool SmartTouch ablation catheter	Biosense Webster (Johnson & Johnson)	Manipulated embolized device in the left ventricle
16-F long arterial sheath	Cordis	Transarterial access for snare retrieval
Perclose sutures	Abbott	Preclosure of the femoral arterial access site
Amplatzer Amulet device	Abbott Laboratories	Abbott Laboratories

LAAO = left atrial appendage occlusion.

## Follow-Up

The patient was evaluated at 1 week, 4 weeks, and 6 months postprocedure, and she was maintained on anticoagulation therapy while being monitored for hemoglobin levels. Later, she underwent a successful Amplatzer Amulet device (Abbott Laboratories) placement without complications. The device was chosen given the appendage's wider ostium and borderline depth. Its lobe and disc design allowed for more stable anchoring compared with the depth-dependent anchoring of the Watchman device.

## Discussion

An LAAO device is widely recognized as an effective alternative to oral anticoagulation in atrial fibrillation, particularly in patients with an elevated risk of bleeding. Although rare, inadvertent device embolization is a known, potentially life-threatening complication of the procedure.^2^ According to the National Cardiovascular Data Registry's LAAO Registry, 30 embolizations (0.07%) were reported between 2016 and 2021. Systematic reviews have shown the incidence of device embolization to range from 0% to 2% of all procedures.^3^^,^^4^ One-third of these cases occurred early, during the procedure itself, while the remaining two-thirds occurred postprocedure, during hospitalization.^3^ Clinical presentations vary widely, from asymptomatic to severe cardiogenic shock. For example, Aminian et al^5^ described a case presenting 1 month postprocedure with acute pulmonary edema and cardiogenic shock due to embolization of the Amplatzer cardiac plug into the left ventricle. In another case, Bhagat et al^6^ reported the asymptomatic delayed embolization of a Watchman FLX device, diagnosed via follow-up transthoracic echocardiography 35 days after implantation.

Regular practice of PASS criteria has been shown to reduce embolization events. In the current case, the device was secured properly using PASS criteria, but it embolized within a short period. Several factors may contribute to device migration, including improper sizing, suboptimal placement, and vigorous tug testing.^1^ The most common embolization sites include the descending aorta (30%), LA (24%), and LV (20%).^4^ Although prompt device removal is crucial, the embolization site significantly influences the choice of retrieval technique and the associated procedural morbidity. Percutaneous retrieval is the preferred approach for devices embolized to the aorta or LA. However, percutaneous extraction from the LV has traditionally been considered unfeasible given the risk of injury to the surrounding structures, particularly the mitral and aortic valves.^7^ Consequently, surgical extraction from the LV is often required, although it carries a risk of significant complications.

Few cases of LAAO device embolization and subsequent retrieval have been reported. Ferraris et al^8^ described a case involving embolization to the atrial side of the mitral valve, which was successfully retrieved percutaneously using a double-snare technique. In a literature review, Aminian et al^9^ found that 80% of reported Watchman device embolizations to the LV were managed surgically. Since then, there has been a rise in the number of emergent surgical retrievals. For instance, Birmingham et al^7^ documented a case of mitral valve embolization that required emergent open heart surgery after multiple failed percutaneous attempts and 2 episodes of pulseless electrical activity. Additionally, Ceresa et al^10^ recently described a case of intraoperative device embolization to the ventricular side of the anterior mitral leaflet, which required surgical intervention after failed percutaneous snare retrieval.

To date, a few successful percutaneous retrievals of embolized LAAO devices from the LV have been reported. Spilias et al^1^ described a case in which an LAAO device was retrieved from the LV using a transarterial approach and a single snare after failed attempts via a transseptal route. In our case, the LAAO device was found wedged between the lateral wall and the papillary muscle. Our strategy involved gentle manipulation to free the device from the surrounding structures, effectively converting a complex LV embolization into a simple embolization. The EN Snare system, equipped with a triple-loop snare offering a higher probability of foreign body capture compared with a single-loop design, was positioned in the descending aorta to safely retrieve the device once it moved from the LV into the aorta.

We describe a novel percutaneous approach that successfully avoided major surgery, its associated complications, and prolonged postoperative recovery. The previously published peer-reviewed articles on embolized LAAO devices are summarized in Supplemental Table 1.

## Conclusions

Device embolization is a rare but life-threatening complication of LAAO. Although retrieval from the aorta and LA can be percutaneous, a surgical approach is often required for LV device entrapment. This case study demonstrates that a percutaneous snare technique can be a viable and safe approach for retrieving an embolized LAAO device from the LV in centers with limited access to cardiothoracic surgery. Notably, the use of triple-snare loops played a key role in securing and extracting the device effectively, providing enhanced control and stability during retrieval. Although the use of multiple-snare loops appears effective, further research is necessary to fully evaluate the safety, potential complications, and procedural nuances. Continued study will help refine the technique and guide its broader application in future cases.

**Visual Summary:**
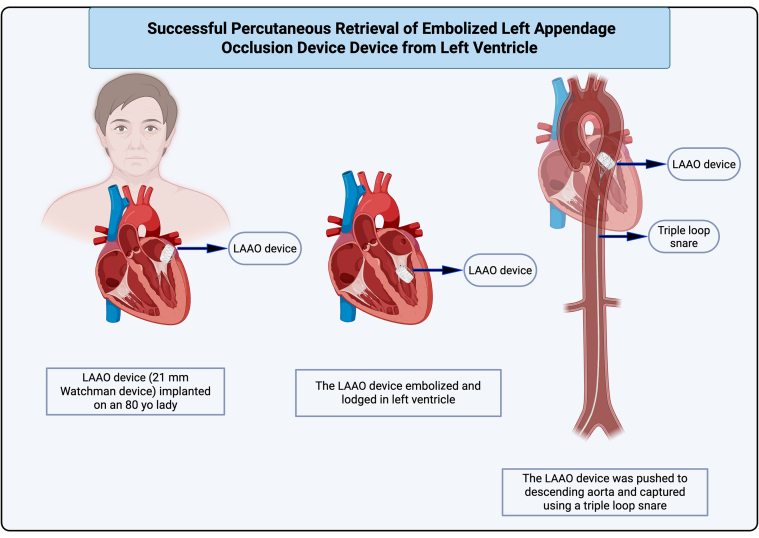
Illustration of Case Presentation The images were created using Biorender.com.

## Funding Support and Author Disclosures

The authors have reported that they have no relationships relevant to the contents of this paper to disclose.

## Appendix

Supplemental Table 1 Published Peer-Reviewed Studies on Embolized LAAO Devices
