# Supplementary material for: Successful Percutaneous Retrieval of Embolized Left Atrial Appendage Occlusion Device From Left Ventricle
Source: JACC Case Rep. 2025 Dec 10;30(40):105902. doi: 10.1016/j.jaccas.2025.105902 (PMC12861665; doi:10.1016/j.jaccas.2025.105902)
Supplement: Supplemental Table 1 — Published Peer-Reviewed Studies on Embolized LAAO Devices [file mmc1.docx]

| Author | Year | Study Type | Age | Gender | Symptoms | Device Type | Embolization Site | Time to Embolization | Retrieval Method |
| --- | --- | --- | --- | --- | --- | --- | --- | --- | --- |
| Alkhouli et al. | 2019 | Review |  |  |  | Amplatzer, Watchman | Various | 12 months | Snare (multiple types) |
| Chopra et al. | 2019 | Case report | 86 | Male | Asymptomatic | Amulet | Left Atrium | 18 hours | Triple loop snare after goose neck failure |
| Spilias et al. | 2019 | Case report | 79 | Male | AF with RVR, hypoxia | Watchman | LV (mitral apparatus) | 6 hours | One-snare device |
| Mansour et al. | 2020 | Case report | 77 | Male | Strokes, foot pain | Watchman | Abdominal aorta | 18 months | No removal due to risk |
| Halim et al. | 2020 | Case report | 77 | Male | Intra-procedural embolization | Watchman | Left Atrium | Immediate | Double guidewire snare |
| Afzal et al. | 2020 | Retrospective |  |  |  | Watchman | Various | Median 45 days | Snare, surgery, forceps |
| Turagam et al. | 2020 | Case series | Various | Mixed | Variable | Watchman, Amulet | Various | 3 months | Raptor, EN Snare, biopsy forceps |
| Murtaza et al. | 2021 | Systematic Review | 75 | Mostly Male |  | ACP, Watchman FLX, Amulet | LA, Aortic valve | Not specified | Snare or surgery depending on location |
| Bogunovic et al. | 2021 | Case report | 80 | Male | Asymptomatic | Amplatzer | Aortic Arch | Post-procedure TEE | Snare catheter |
| Ferraris et al. | 2022 | Case report | 75 | Male | Asymptomatic | Watchman FLX | Mitral valve plane | 3 months | Osypka lasso snare |
| Roberts et al. | 2022 | Case report | 79 | Female | Shock | Watchman FLX | LA → LV → Aortic valve | During procedure | Surgical removal |
| Ceresa et al. | 2023 | Case report | 76 | Male | Instability | Watchman FLX | LV, anterior mitral leaflet | Immediate | Failed snare, surgery |
| Horton et al. | 2023 | Case report | 73 | Male | Pain, neuro deficits | Watchman | Infrarenal Aorta | 6 hours | Surgical removal |
| Friedman et al. | 2023 | Retrospective |  | Mostly Male |  | Watchman | Various | 45-day FU | Surgical |
| Bhagat et al. | 2023 | Case report | 73 | Male | Asymptomatic | Watchman FLX | Mitral valve → LVOT | 45 days | Surgical |
| Eppinger et al. | 2024 | Retrospective | 74 | Mostly Male | Variable | Amulet, Watchman | Aorta, LV, LA, periphery | <24h common | Snare, forceps, and surgery |
| Boa et al. | 2024 | Case report | 72 | Male | Asymptomatic | LACbes | Left Atrium | 24 hours | Goose neck snare, forceps |
| Abbadi et al. | 2024 | Case report | 85 | Male | SOB | Amulet | LV (mitral apparatus) | Few hours | Gooseneck snare |
| Wong et al. | 2024 | Case report | 69 | Male | Asymptomatic | Watchman FLX | Left Atrium | 10 weeks | Raptor forceps, snare |
| Goodyear et al. | 2025 | Case report | 77 | Male | Asymptomatic | Watchman FLX | LVOT near the mitral leaflet | 45 days | Rat tooth/alligator forceps |

**Supplemental Table 1:** This table summarizes published peer reviewed literature on embolized left atrial appendage occlusion devices. The device type, site of embolization, and this table summarizes reported cases, series, and reviews concerning embolized left atrial appendage occlusion (LAAO) devices. The patient demographics, symptoms, device types, embolization sites, time to embolization, and retrieval methods were described.
